# Thermal amplification by large-footprint buildings in cold-climate cities: Implications for urban heat mitigation

**DOI:** 10.1016/j.buildenv.2026.114345

**Published:** 2026-03-09

**Authors:** Victoria Miles, Igor Esau, Vera Kuklina

**Affiliations:** a Nansen Environmental and Remote Sensing Center/ Bjerknes Center for Climate Research, Jahnebakken 3, 5007 Bergen, Norway; b The Arctic University of Norway, PO Box 6050 Stakkevollan, NO-9037 Tromsø, Norway; c The George Washington University, 2025 F Street, NW, Washington, D.C. 20052, USA

**Keywords:** Urban heat, Local climate zone (LCZ), Cold-climate cities, Land surface temperature (LST), Building morphology, Thermal environment, Arctic urbanism, Climate-responsive design

## Abstract

The optimal architectural paradigm for human settlements in cold climates remains actively debated, with large-footprint, low-rise buildings often promoted as protection against extreme environmental conditions. However, the broader impact of such developments on urban thermal anomalies in Arctic and sub-Arctic cities remains poorly quantified. This study evaluates the city-scale thermal influence of large low-rise structures classified as Local Climate Zone 8 (LCZ 8) in Fairbanks (USA), Tromsø (Norway), and Nadym (Russia), three high-latitude cities with contrasting urban morphologies. Using Landsat thermal infrared imagery, refined OpenStreetMap building footprints, and GIS-based spatial and statistical analyses, we show that expansive low-rise buildings (>10,000 m^2^ footprint) consistently act as localized summer thermal hotspots. Hotspot intensity and spatial clustering increase systematically with building footprint size and spatial aggregation, with land surface temperatures up to 4°C higher than surrounding urban areas. These anomalies are associated with extensive impervious and dry surfaces, flat roofs, and limited vegetation, which enhance solar absorption and thermal inertia, particularly under weak atmospheric mixing conditions characteristic of cold-climate urban environments. The results challenge conventional urban heat island assumptions by demonstrating that horizontal building scale is a key control on urban thermal behavior in cold regions. Importantly, these findings do not imply that northern cities require heat adaptation strategies derived from warm-climate contexts. Instead, they highlight the need to re-evaluate how localized warming interacts with infrastructure performance, seasonal comfort, and outdoor activity patterns, positioning LCZ 8 districts as priority zones for climate-responsive and seasonally adaptive urban design.

## 1. Introduction

Urban development in cold and high-latitude regions poses persistent challenges for planning, environmental performance, and human comfort. Arctic and sub-Arctic cities are shaped by long winters, short summers, and strong seasonal contrasts that influence both urban form and everyday activity patterns. In response, debates on sustainable urbanization in cold climates have historically revolved around two contrasting architectural paradigms [1,2]. One paradigm, often characterized as “a city under the dome” and informed by Ralph Erskine’s thinking on northern settlements [3], envisions urban life being largely shielded from harsh outdoor conditions through compact, predominantly enclosed building complexes. In contrast, the paradigm of a “city embracing cold climates” argues for continued engagement with outdoor space, where urban form and public space are designed to function despite climatic constraints rather than avoiding them altogether [1,4]. Although these paradigms differ in their attitudes toward outdoor exposure, both rely heavily on large-footprint buildings, whose impacts on surrounding outdoor thermal environments have historically received limited quantitative attention, as urban climate research in high latitudes focused primarily on indoor comfort and winter performance [1,5].

The advent of satellite-based thermal remote sensing has fundamentally changed the study of urban climate in high latitudes. Land surface temperature (LST) products enable systematic observation of urban thermal environments across spatial scales, revealing how urbanization modifies surface energy balances and environmental quality [6]. Large-scale analyses demonstrate that surface urban heat island effects are a widespread feature of cities globally [7–9], and recent studies confirm that summer surface temperature anomalies are robust and spatially structured even in Arctic and sub-Arctic cities [5,10–12]. In cold climates classified as Köppen–Geiger ET, Dfb, and Dfc, limited incoming radiation, weak convection, and stable stratification suppress vertical mixing, allowing surface temperature anomalies to remain spatially confined and persist near their source, making surface temperature anomalies sharper and more localized than in warmer regions [7,13,14].

Within this climatic context, urban morphology exerts a strong control on surface thermal behavior. The Local Climate Zone (LCZ) framework provides a standardized classification linking urban form, surface cover, and function to characteristic thermal responses (Stewart & Oke, 2012) [15,16]. Developed within the WUDAPT initiative, LCZs enable comparative analysis of urban thermal environments using globally consistent remote-sensing–based data and have been successfully applied across a range of climatic settings, including high-latitude cities [7,15–20].

However, LCZ-specific thermal behavior in Arctic and sub-Arctic cities remains insufficiently resolved, particularly for large low-rise buildings classified as LCZ 8. LCZ 8 is characterized by horizontally extensive building footprints, low building heights, high impervious surface fractions, and sparse vegetation [15]. In cold-climate cities, this class commonly includes industrial areas, logistics hubs, airports, retail complexes, and large institutional facilities—urban forms that are spatially extensive and functionally important yet underrepresented in detailed LCZ-focused thermal analyses.

The prominence of LCZ 8 in high-latitude cities reflects both long-standing environmental adaptation and recent development trends. Northern urban areas have traditionally favored low- to medium-height, horizontally extensive buildings to reduce wind exposure, limit heat loss, and accommodate permafrost-related constraints [21–23]. These principles were institutionalized in Soviet-era Arctic cities through standardized building forms [24,25] and have been further reinforced in recent decades by extensive non-residential developments—such as retail complexes, logistics facilities, and transport infrastructure—often adopting design logics optimized for milder climates [26]. Together, these processes have produced large, spatially continuous LCZ-8 districts with high impervious surface fractions, fundamentally altering land-–atmosphere interactions [27,28].

During summer, LCZ-dependent thermal behavior differs markedly between climate regions. In temperate and tropical cities, compact high-rise zones (LCZ 1–2) typically exhibit the highest surface temperatures due to dense building clusters and limited ventilation [29–31]. In cold-climate cities, however, surface imperviousness and horizontal extent often exert stronger control on surface warming than building height, making LCZ 8 a potentially dominant contributor to summer surface temperature anomalies [32].

Evidence from LCZ-based analyses and in situ observations indicates that LCZ 8 areas in cold-climate cities frequently coincide with pronounced summer thermal hotspots. Moreover, the contrast between LCZ 8 and other urban zones appears stronger in cold climates than in warmer regions, consistent with reduced atmospheric mixing and limited evapotranspiration. In situ observations in Arctic cities, such as Nadym, Russia, demonstrate strong localization of surface temperature anomalies over large-building and industrial districts, linked to stable stratification and weak turbulence [33,34]. These findings suggest that LCZ 8 morphology may exert a primary control on summer surface temperature extremes in high-latitude cities.

This study addresses this gap by systematically investigating summer surface temperature anomalies effects associated with spatially continuous LCZ-8 districts formed by large-footprint, low-rise building projects in Arctic and sub-Arctic cities. Focusing on Fairbanks (USA), Tromsø (Norway), and Nadym (Russia), we use Landsat thermal infrared imagery, refined OpenStreetMap building footprints, and spatial hotspot analysis to: (1) quantify the contribution of aggregated large-footprint, low-rise building projects to district-scale summer LST anomalies within LCZ-8 areas; (2) evaluate how building footprint size and spatial aggregation influence the intensity and clustering of LCZ-8 thermal hotspots; and (3) discuss the implications of the observed LCZ-8 thermal behavior for climate-responsive urban heat mitigation and planning in cold-climate environments.

## 2. Materials and methods

### 2.1. Study area

**Fig. 1.**
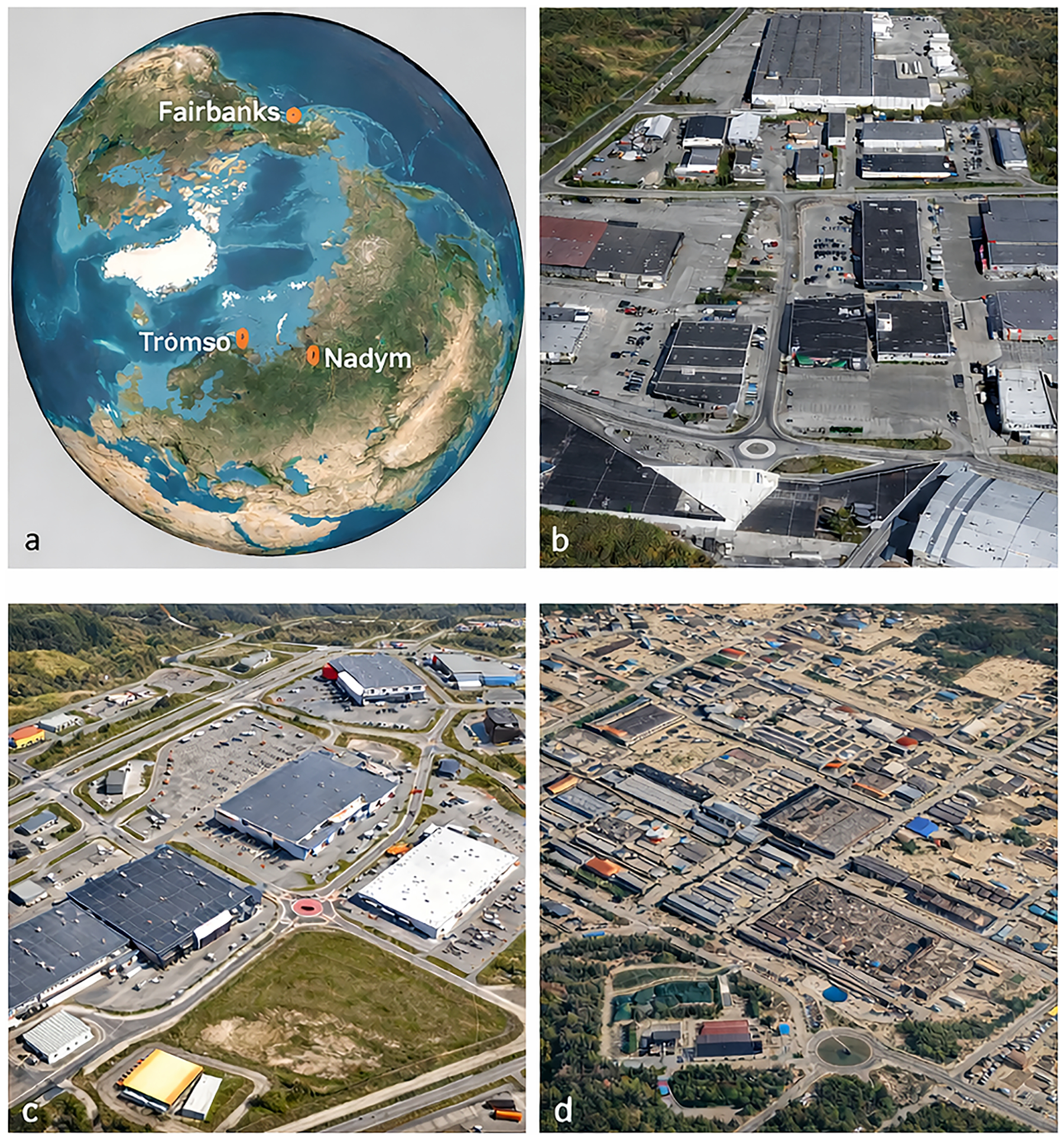
Location of the three case study cities—Fairbanks (USA), Tromsø (Norway), and Nadym (Russia). Insets show representative large-footprint, low-rise buildings such as shopping centers, warehouses, and industrial complexes.

We investigate urban thermal patterns in three truly cold-climate Arctic cities: Fairbanks (USA), Tromsø (Norway), and Nadym (Russia) (Fig. 1). All three cities are located in the high latitudes and experience subarctic climates classified as Köppen–Geiger Dfc. Despite their similar climatic settings, they differ markedly in urban morphology, ranging from the sprawling continental layout of Fairbanks to the compact, maritime configuration of Tromsø and the centrally planned grid structure of Nadym. The three cities are not intended to be statistically representative of all Arctic or sub-Arctic urban environments. Instead, Fairbanks, Tromsø, and Nadym are selected as contrasting archetypes of cold-climate urban form, reflecting different development histories, planning traditions, and spatial configurations. This comparative design allows us to test whether LCZ-8-related thermal behavior is robust across diverse cold-climate urban contexts rather than to infer universal temperature magnitudes.

The prevalence of large-footprint, low-rise buildings in Fairbanks, Tromsø, and Nadym reflects not only climatic constraints but also distinct historical, cultural, and planning traditions. In Fairbanks, automobile-oriented development and dispersed commercial zoning favor horizontally extensive structures; in Tromsø, recent growth has promoted compact but large institutional and commercial complexes within topographic constraints; and in Nadym, Soviet-era planning emphasized standardized, spatially concentrated building forms adapted to permafrost conditions. These differing development pathways result in comparable LCZ-8 morphologies despite contrasting governance and cultural contexts. Together, these cities provide a comparative framework for examining how urban form modulates summer land surface temperature (LST) patterns under similar cold-climate conditions.

Fig. 1 highlights a recurring building typology across all three cities: large-footprint, low-rise structures characterized by extensive impervious surfaces, flat roofs, and limited vegetation. Despite differing planning traditions, the consistent presence of these LCZ-8 morphologies across Fairbanks, Tromsø, and Nadym motivates their use as focal units for district-scale LST analysis.

Fairbanks, USA (64°50′N, 147°43′W; ~71,000 inhabitants), is in interior Alaska and experiences an extreme continental subarctic climate with very cold winters (mean winter temperatures below −20°C) and relatively warm summers (mean summer temperatures around 17°C). Urban development is strongly influenced by permafrost constraints and is characterized by a low-density, sprawling layout dominated by detached housing. Large-footprint, low-rise buildings are common in commercial, industrial, and airport zones, producing extensive LCZ-8 areas comparable to those found in many North American cities.

Tromsø, Norway (69°40′N, 18°56′E; ~70,000 inhabitants), is a coastal city with a subarctic maritime climate moderated by the North Atlantic Current. Urban development is compact and topographically constrained, with most construction concentrated on Tromsøya Island. Recent population growth has spurred the development of large, low-rise complexes, including shopping malls, logistics facilities, and airport-related infrastructure, typical of LCZ-8 morphology, particularly along the western part of the island.

Nadym, Russia (65°32′N, 72°31′E; ~50,000 inhabitants), is in the continental interior of western Siberia and experiences a severe subarctic climate with long, cold winters and widespread permafrost. The present city, developed primarily during the Soviet period, occupies a compact area of approximately 6 km^2^ and is characterized by mid-rise residential blocks arranged in regular grids with centralized heating systems, corresponding largely to LCZ 5. Urban expansion beyond the core area is limited, vegetation cover is sparse, and built infrastructure is adapted to permafrost conditions, making Nadym representative of many mono-industrial cities in the Russian Arctic.

**Fig. 2.**
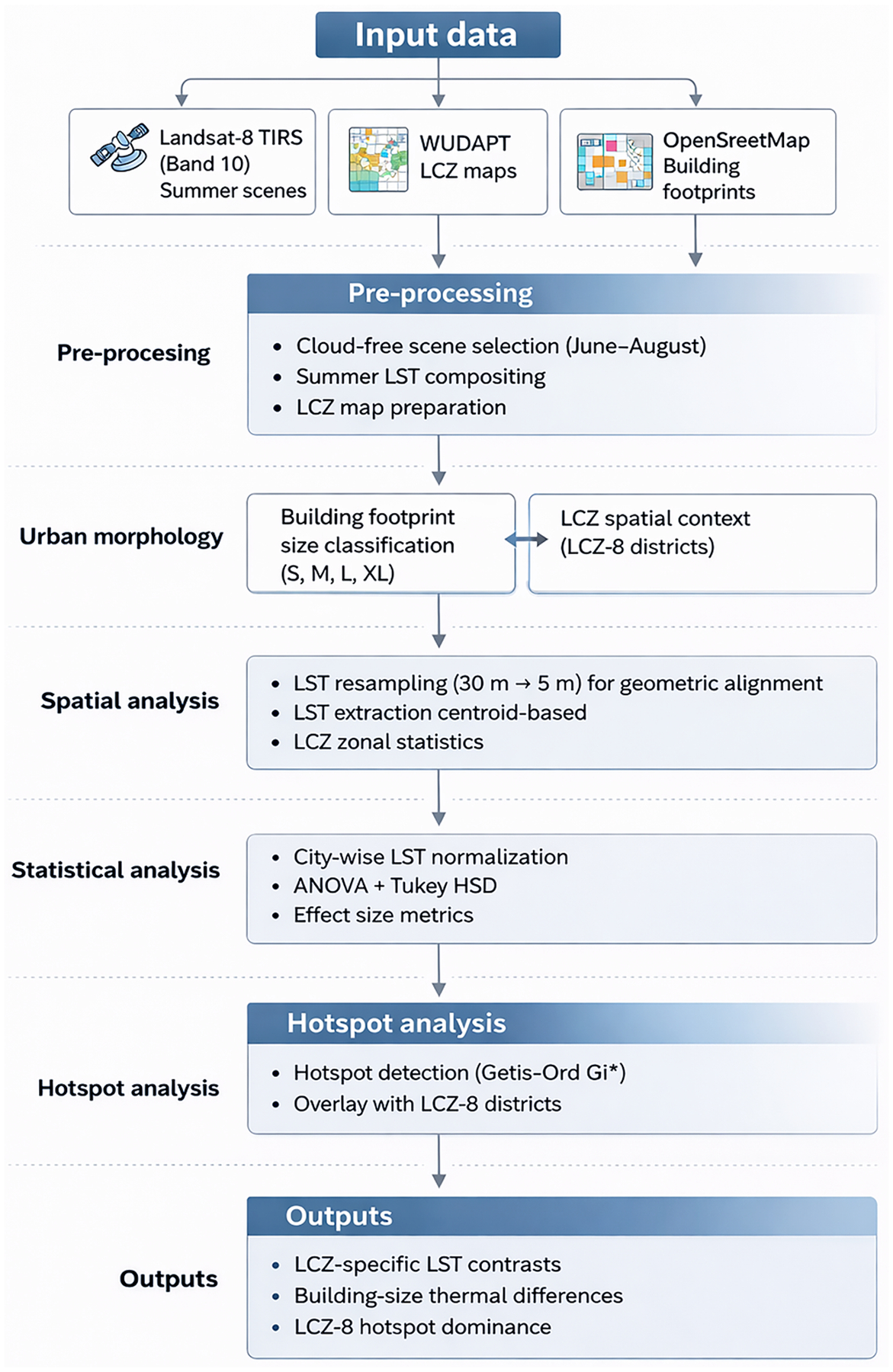
Methodological workflow of the study.

Together with Fairbanks and Tromsø, Nadym provides a contrasting cold-climate urban morphology for evaluating the thermal effects of large-footprint development. Although residential and commercial energy-use practices differ among Fairbanks, Tromsø, and Nadym, this study focuses on summer clear-sky conditions when space-heating demand is minimal; therefore, observed land-surface temperature patterns primarily reflect surface form and cover rather than heating-related anthropogenic heat. Fig. 2 summarizes the data sources, preprocessing steps, and analytical workflow used to ensure consistent comparison across the three study cities. Fig. 2 provides an overview of the data sources, preprocessing steps, and methodological workflow used in this study.

### 2.2. Remote sensing data

Land surface temperature (LST) data were derived from imagery captured by the Thermal Infrared Sensor (TIRS) onboard Landsat-8. The TIRS instrument acquires thermal data at a native spatial resolution of 100 m, which is resampled to 30 m to match the Landsat multispectral bands, enabling district-scale urban analysis. In accordance with U.S. Geological Survey (USGS) [35] recommendations to avoid calibration uncertainties associated with split-window approaches, only Band 10 (10.60–11.19 μm) was used in this study.

LST was retrieved using a standardized single-channel methodology widely applied in urban climate studies, e.g., Avdan & Jovanovska [36]. The processing included: (1) conversion of raw digital numbers to top-of-atmosphere radiance, (2) calculation of at-sensor brightness temperature, and (3) correction for surface emissivity. Surface emissivity was estimated using an NDVI-based method following established practice for heterogeneous urban surfaces. No independent, scene-specific atmospheric correction was applied beyond the standard processing embedded in the Landsat data products, consistent with the study’s focus on relative spatial contrasts rather than absolute temperature retrieval.

Landsat-derived LST is subject to uncertainties related to sensor noise, atmospheric effects, and emissivity estimation. Previous validation studies have shown that Landsat-8 Band-10 LST typically exhibits biases below 1 K and root-mean-square errors of 1–3 K under clear-sky conditions [37–41]. Accordingly, the present analysis focuses on relative spatial differences in LST across building size classes and LCZ districts, which substantially exceed these uncertainty bounds and are therefore robust for comparative assessment.

Nine cloud-free Landsat-8 scenes (three per city) acquired during June–August between 2013 and 2025 were analyzed (Table 1). The temporal range reflects the operational period of Landsat-8, launched in 2013, with 2025 representing the most recent data available at the time of analysis. Each scene was visually inspected to exclude clouds, haze, and other artifacts. For each city, the three scenes were averaged to produce a representative summer LST composite, reducing synoptic variability while preserving stable spatial thermal patterns. We note that the analysis is not intended to represent full seasonal or diurnal thermal dynamics or to assess heat exposure or health risks. Instead, the study focuses on persistent spatial contrasts in land surface temperature under comparable mid-summer, clear-sky conditions. Averaging multiple cloud-free summer scenes per city reduces short-term meteorological noise while preserving morphology-driven spatial patterns.

### 2.3. LCZ mapping

The LCZ maps for Tromsø, Fairbanks, and Nadym were downloaded from the World Urban Database and Access Portal Tools (WUDAPT), which provides standardized global classifications following the Local Climate Zone (LCZ) framework [15,18,19]. The WUDAPT Level-0 method applies supervised classification of satellite imagery to delineate LCZ classes, offering consistency across regions and facilitating inter-city comparisons.

WUDAPT has certain limitations. Its automated classification relies on medium-resolution imagery and two-dimensional training areas, which do not account for building height or finer morphological differences. As a result, this method often oversimplifies heterogeneous urban environments, particularly in smaller cities [17,18]. In the case of Nadym, the WUDAPT classification labelled the entire urban area as LCZ 8, thereby obscuring the diversity of both its urban and natural structures.

To improve accuracy, we manually refined the Nadym LCZ map using ArcGIS, incorporating building footprints from OpenStreetMap and high-resolution base maps. This refinement led to the identification of additional LCZ types, including LCZ 5 (Open mid-rise), LCZ 6 (Open low-rise), LCZ 9 (Sparsely built), LCZ A (Dense trees), and LCZ E/F (Bare rock). As a result, we achieved a more precise representation of the city’s morphology. Tromsø and Fairbanks were retained in their WUDAPT form without modification.

### 2.4. Urban morphology and building categorization

Building footprint data were obtained from OpenStreetMap and classified into four categories based on horizontal area: small (<1,000 m^2^), medium (1,000–5,000 m^2^), large (5,000–10,000 m^2^), and extra-large (>10,000 m^2^). These thresholds were selected to capture distinct building forms with characteristic thermal behaviors, serving as a proxy for LCZ related to surface structure and material properties [15]. The categorization leverages OpenStreetMap building footprints, which are widely used in large-scale urban analyses and support consistent cross-city comparisons of building form [42,43].

This categorization highlights the building forms most relevant to cold-climate urbanism. The large and extra-large categories correspond to LCZ 8, encompassing expansive industrial complexes, shopping malls, and institutional facilities with extensive impervious cover. Such structures are strongly associated with elevated LST due to their vast surface area for absorbing solar radiation and limited shading [44,45].

In contrast, the small and medium categories represent the background urban fabric, typically aligned with LCZ 5 and LCZ 6. These areas usually exhibit lower impervious surface fractions and greater vegetation cover, which contribute to cooler surface conditions [46–48].

Table 2 summarizes the number of buildings in each category for Fairbanks, Tromsø, and Nadym. The groups are naturally unbalanced—small and medium buildings dominate numerically across all Arctic cities—yet each class contains a sufficiently large sample for stable statistical estimation. The smaller number of extra-large buildings is not a sampling artifact but an inherent morphological property of urban form: structures exceeding 10,000 m^2^ are physically large, often occupying an entire block, and therefore only occur in limited numbers. This reflects the true composition of the urban fabric rather than any data limitation.

### 2.5. Spatial and statistical analysis

Urban thermal patterns were analyzed using a combination of LCZ-based zonal statistics, building-level LST extraction, and spatial hotspot analysis implemented in ArcGIS Pro 3.2. The analysis focuses on relative thermal differences associated with urban morphology and building footprint size, rather than on retrieving exact roof temperatures for individual buildings.

To characterize district-scale thermal behavior, Local Climate Zone (LCZ) polygons were overlaid on the composite summer LST surfaces. Zonal statistics were used to calculate mean LST values for each LCZ present in the study areas, providing a comparative overview of how standardized urban typologies influence surface temperatures within each city.

To assess the role of building size in shaping surface thermal behavior, all building footprints were assigned to one of four footprint-area categories (small, medium, large, and extra-large; see Section 2.4). For each building, a representative LST value was extracted from the composite LST surface.

Landsat-derived LST has a native spatial resolution of 100 m (distributed at 30 m), which can introduce pixel–building misalignment and mixed-pixel effects when combined with vector building footprints. To address this issue, the 30 m LST surface was resampled to 5 m using bilinear interpolation. This operation does not add new thermal information or increase the effective spatial resolution; rather, it improves geometric alignment between the raster grid and vector footprints, reducing boundary artifacts and stabilizing centroid-based sampling.

LST was sampled at the centroid of each building footprint, a common approach in LCZ–LST studies that minimizes edge effects where roofs, parking areas, and vegetation intersect. This method is particularly appropriate for large and extra-large buildings, which typically span multiple Landsat pixels, such that the centroid falls within a relatively homogeneous impervious surface.

To evaluate the sensitivity of the results to the extraction method, we conducted a robustness test comparing centroid-based LST values with area-weighted LST values calculated as the mean of all pixels intersecting each building footprint. Differences between the two approaches were small (≤0.15°C at the class level), and no ANOVA significance levels or effect-size metrics changed. These results indicate that the reported thermal contrasts are not driven by pixel–building matching artifacts.

Differences in LST among building footprint categories were evaluated using one-way analysis of variance (ANOVA), followed by Tukey’s Honest Significant Difference (HSD) post hoc tests. Because Fairbanks, Tromsø, and Nadym differ in background climate, surface materials, and absolute temperature ranges, building-level LST values were normalized within each city before ANOVA.

For each city *c*, standardized LST values were computed as:

$$z_{i,c}=\frac{LST_{i,c}-\mu_{c}}{\sigma_{c}}$$

Where *LST*_*i,c*_ is the surface temperature of the building *i* in the city *c*, and *μ*_*c*_ and *σ*_*c*_ are the citywide mean and standard deviation of building-level LST. This normalization ensures that statistical results reflect relative intra-urban thermal deviations rather than inter-city climatic differences. Effect-size metrics (η^2^ for ANOVA and Cohen’s *d* for pairwise comparisons) were calculated alongside significance testing to confirm that observed differences are substantial and not driven by unequal sample sizes.

The spatial concentration of thermal extremes was assessed using the Getis–Ord Gi* statistic [49,50]. This method compares each feature’s LST value with those of neighboring features. It produces standardized *z*-scores and associated *p*-values; significant positive *z*-scores indicate hotspots, and negative values indicate cold spots. A fixed Euclidean distance band of 10 m was applied to capture local clustering of building-scale anomalies, ensuring a consistent neighborhood scale across all three cities, and a false discovery rate (FDR) correction (α = 0.05) was used to control for multiple testing. Hotspots were classified using standard confidence bins (±1 = 90 %, ±2 = 95 %, ±3 = 99 %).

To relate thermal clustering to urban morphology, hotspot maps were overlaid with LCZ-8 polygons, and the proportion of hotspot area located within LCZ-8 districts was quantified. Sensitivity tests using alternative distance bands (5–30 m) produced similar hotspot patterns, indicating that the results are robust to reasonable variations in neighborhood definition.

All analyses that report absolute temperature values (LCZ summaries, hotspot mapping, and spatial statistics) were conducted using unstandardized LST to preserve the physical magnitude of surface temperature differences.

## 3. Results and analysis

### 3.1. Thermal patterns and hotspot clustering across LCZs

**Fig. 3.**
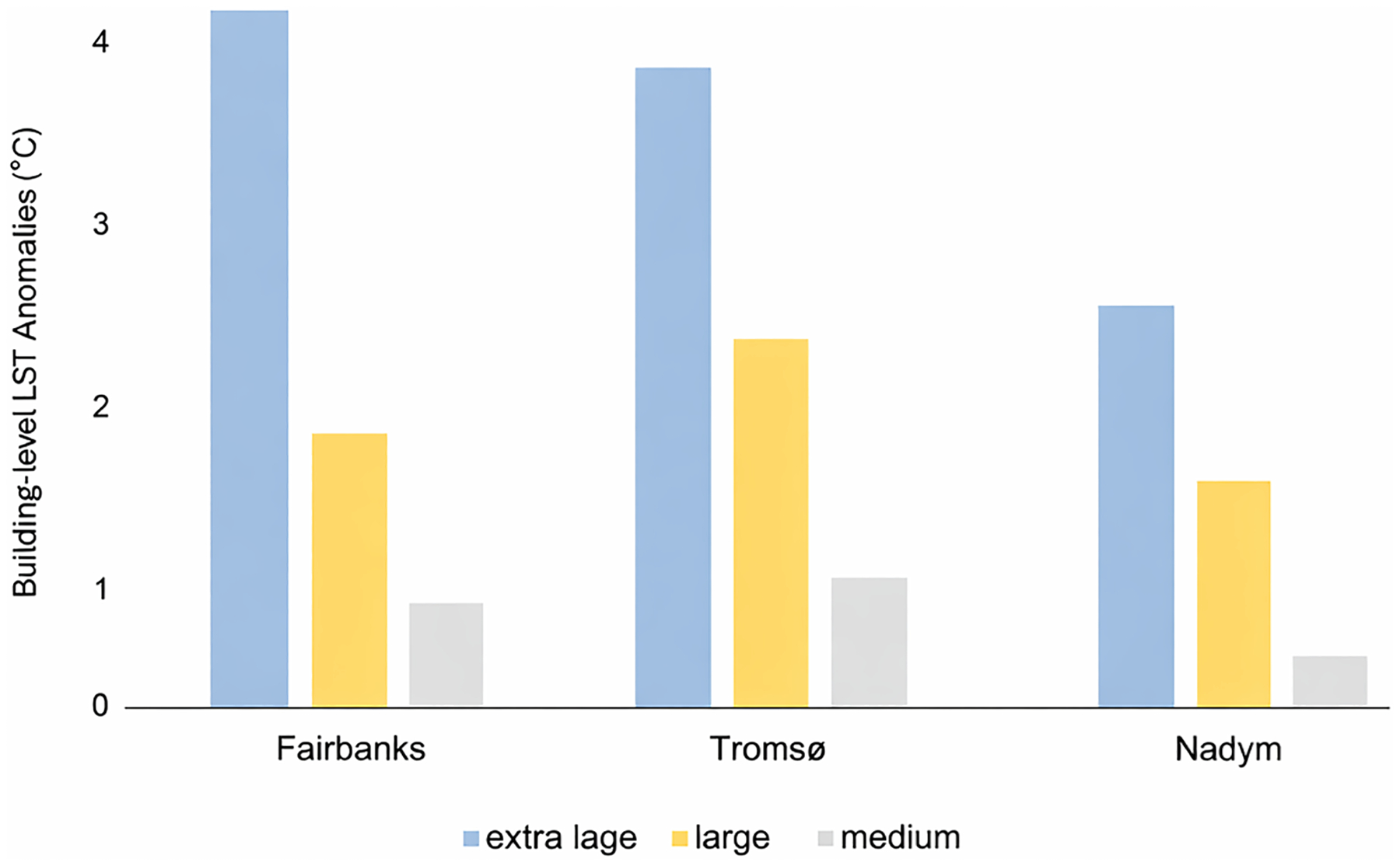
Mean land surface temperature (LST) anomaly by building size and city. Anomalies for medium, large, and extra-large building categories are calculated relative to the small category (baseline = 0.0).

Across all three cities, building footprint size exerts a strong control on summer land surface temperature (LST). Extra-large and large buildings consistently record the highest maximum LSTs, while small and medium buildings form a cooler background urban fabric (Table 3; Fig. 3). In Fairbanks, maximum LST for extra-large buildings exceeds 38°C, compared with 36.8°C for large buildings and 33.7°C for small buildings. Tromsø and Nadym show the same ordering, with extra-large buildings exceeding small buildings by approximately 4–6°C. Thermal variability also increases with footprint size: small, medium, and large buildings exhibit standard deviations of ~1–1.5°C, whereas extra-large buildings reach up to 2.8°C in Fairbanks, indicating heterogeneous microclimates associated with extensive impervious surfaces.

**Fig. 4.**
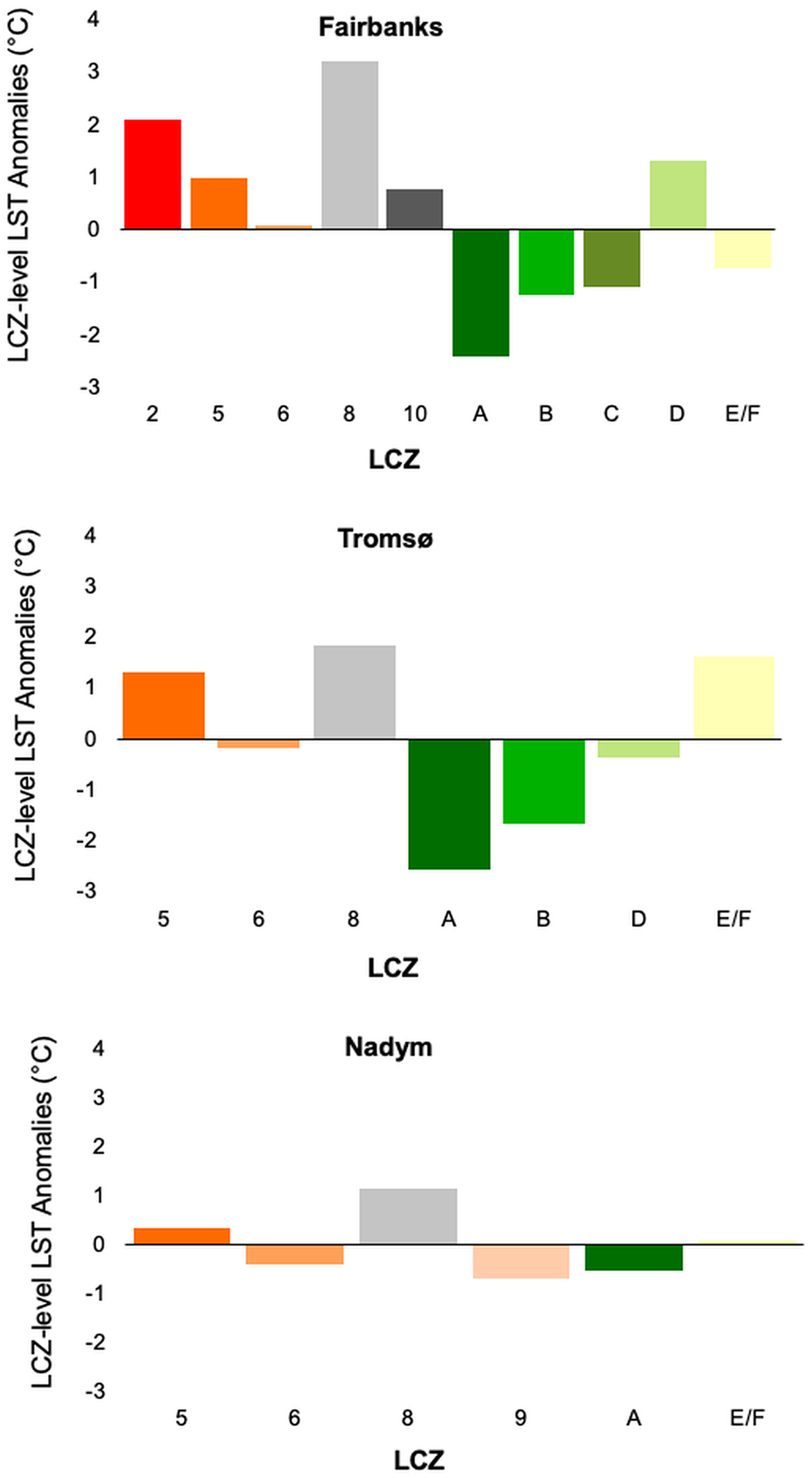
LCZ-level land surface temperature (LST) anomalies relative to the citywide mean for Fairbanks (top), Tromsø (middle), and Nadym (bottom). LCZ 8 consistently exhibits positive anomalies, while vegetated LCZs show negative deviations.

LCZ-level analysis confirms these building-scale patterns. In all three cities, LCZ 8 (large low-rise) exhibits the strongest positive anomalies relative to the citywide mean, whereas vegetated classes (e.g., LCZ A) are consistently cooler by 2–4°C (Table 4; Fig. 4). In Fairbanks, LCZ 8 averages approximately +2.2°C above the city mean, while dense and scattered tree zones are −2.4°C and −1.3°C cooler, respectively (Table 4). Tromsø and Nadym show a similar but weaker contrast, with LCZ 8 around +1.0°C above the mean and tree-covered zones remaining 2–3°C cooler (Table 4).

Getis–Ord Gi* hotspot analysis reinforces the dominance of LCZ 8 in shaping summer thermal extremes. Across all cities, statistically significant hotspots align disproportionately with LCZ-8 polygons, indicating that horizontal extent and impervious surface coverage—rather than building height—govern the spatial concentration of surface heating.

**Fig. 5.**
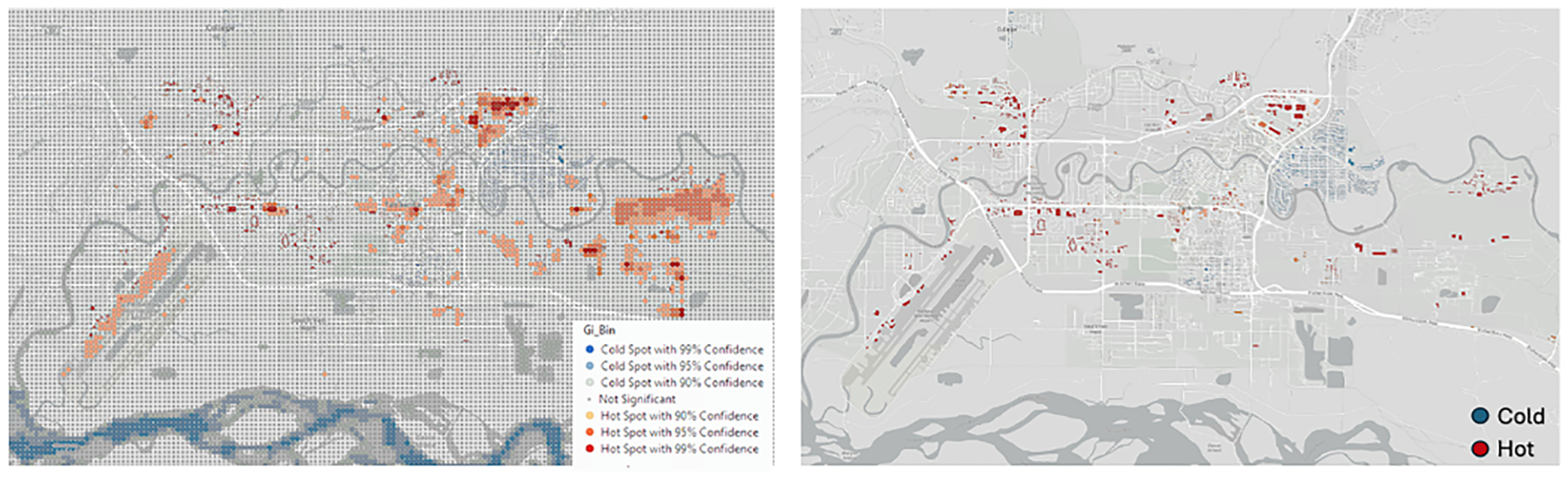
Fairbanks: Getis–Ord Gi* analysis of land surface temperature (LST) hot and cold spots (left) and statistically significant building-based LST hotspots (right). LST hotspots predominantly align with LCZ 8 clusters containing the city’s largest building footprints; however, individual thermally hot buildings may still occur within statistically defined cold-spot areas due to neighborhood-scale spatial clustering rather than individual building temperatures.

In Fairbanks, hotspots appear as fragmented clusters rather than a single continuous zone (Fig. 5). LCZ 6 and LCZ 8 occupy comparable spatial extents. Still, the strongest hotspot clusters align with LCZ 8 and parts of LCZ 5, while LCZ 6 residential areas exhibit a mixed pattern with the highest share of coldspots. Building-LCZ statistics show that LCZ 8 has the largest mean footprint (711 m^2^), nearly three times that of LCZ 5 and LCZ 6, with 29 % of buildings classified as large or extra-large (Table 5). Correspondingly, LCZ 8 and LCZ 5 record hotspot shares close to 90 %, whereas LCZ 6 exhibits substantially more coldspots (Table 6).

**Fig. 6.**
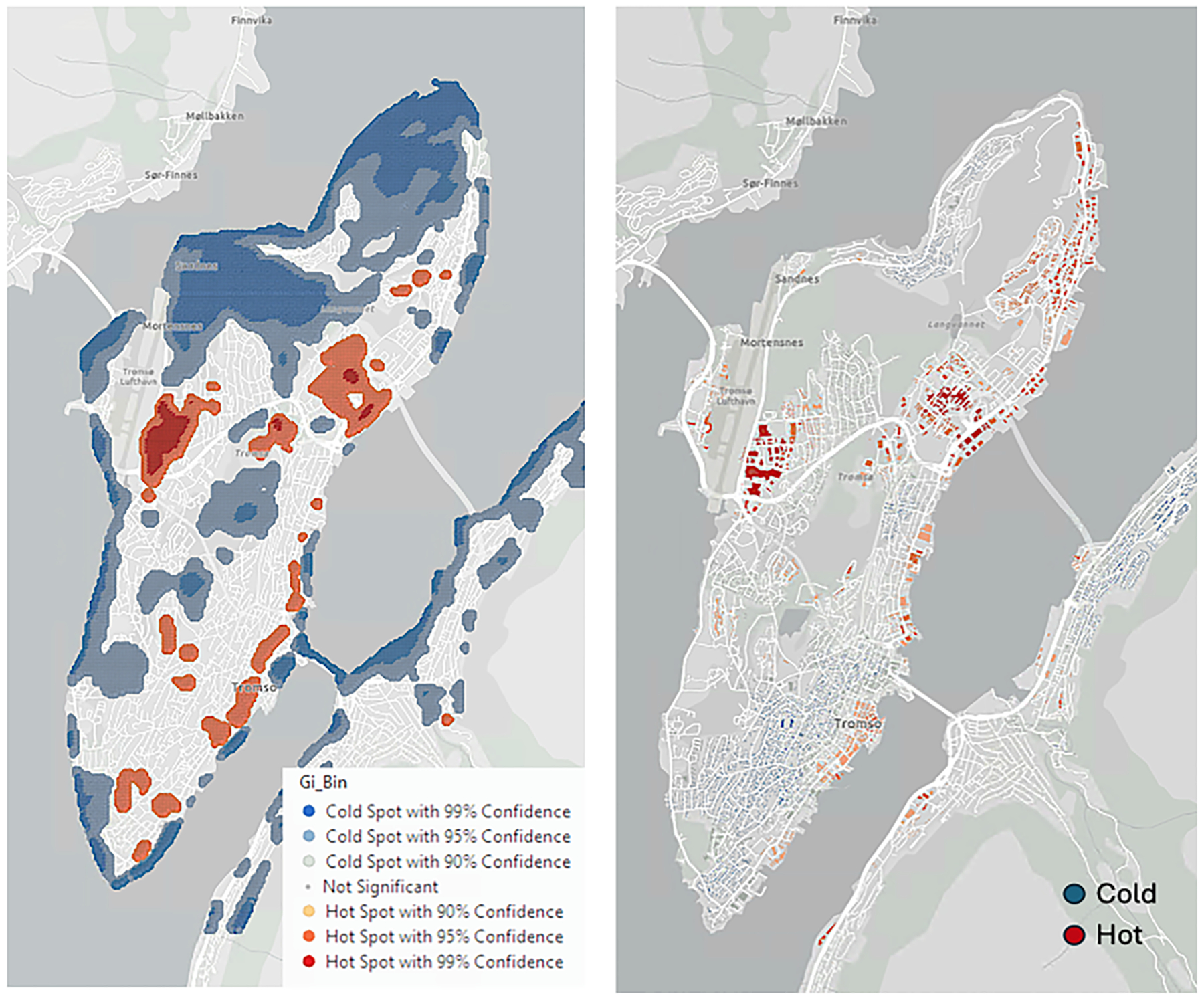
Tromsø: Getis–Ord Gi* analysis of land surface temperature (LST) hot spots (left) and statistically significant building-based LST hotspots (right). Hotspots cluster primarily within LCZ 8 and LCZ 2/3 zones, while cooler pathways are evident in LCZ A and LCZ G areas.

In Tromsø, hotspot clusters are spatially concentrated in the compact urban core and align primarily with LCZ 8 and LCZ 2 (Compact midrise) (Fig. 6). LCZ 8 forms the morphological core in footprint terms, with a mean building area of 978 m^2^ and more than 40 % of buildings classified as large or extra-large (Table 7). Hotspot statistics confirm LCZ 8 as the dominant hotspot zone, with approximately 41 % of buildings classified as hotspots and only 11 % as coldspots, compared with cooler, residential LCZ 6 areas (Table 8).

**Fig. 7.**
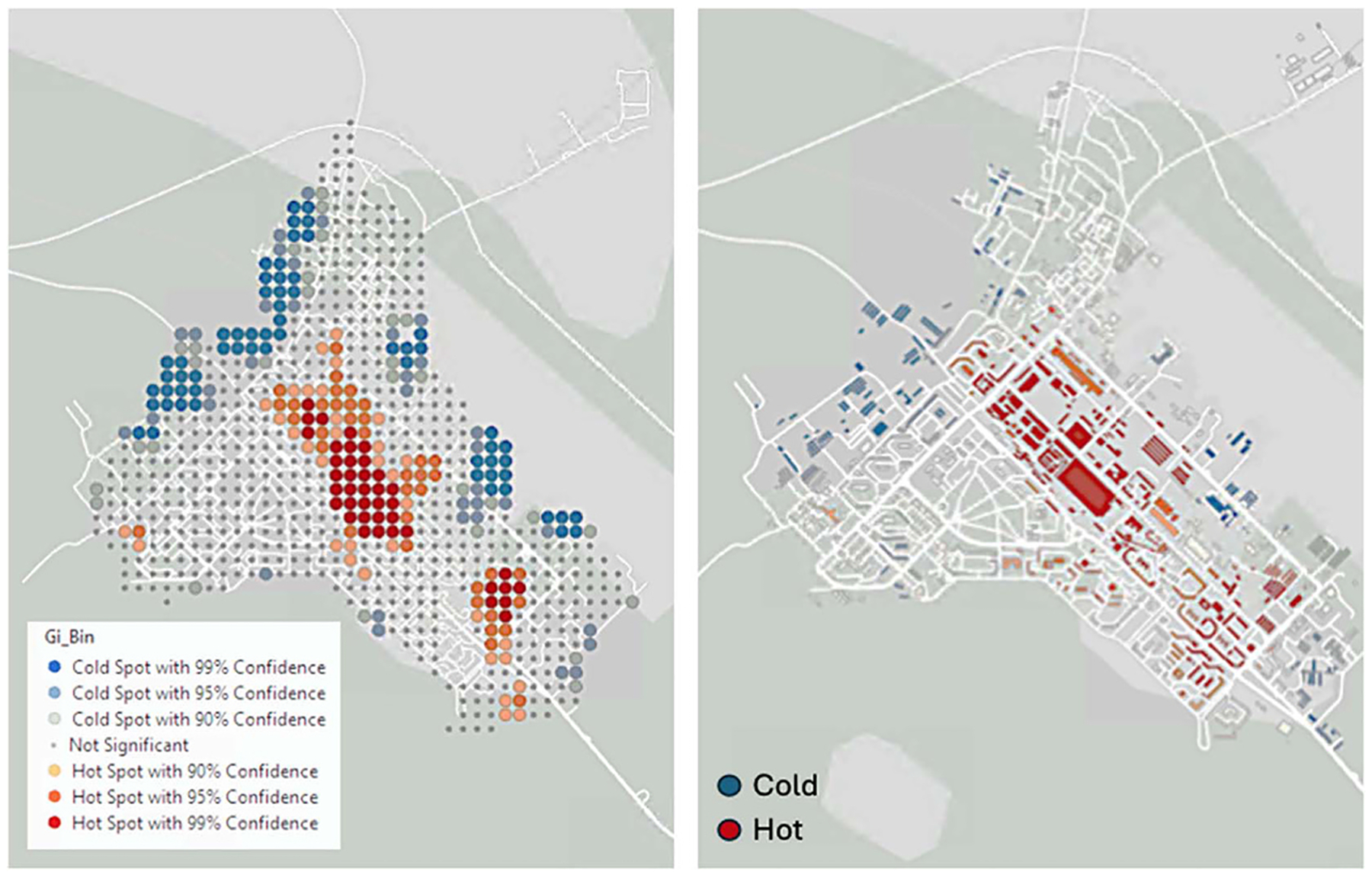
Nadym: Getis–Ord Gi* analysis of land surface temperature (LST) hot spots (left) and statistically significant building-based LST hotspots (right). Intra-urban variation highlights LCZ 8 as the dominant hotspot zone.

In Nadym, the built-up area is markedly warmer than the surrounding tundra and forest (Fig. 7). Within the city, hotspot clusters are concentrated in the central urban–industrial core, dominated by LCZ 8, while peripheral LCZ 5 and LCZ 6 areas are cooler and more heterogeneous; vegetated zones remain consistently cool. LCZ 8 exhibits the largest mean building footprint (1,432 m^2^) and has more than 60 % of buildings classified as large or extra-large (Table 9). Correspondingly, LCZ 8 records the highest hotspot share (83 %) and the highest mean LST (25.0°C), exceeding all other urban classes (Table 10).

Taken together, maximum LST values, anomaly profiles, and hotspot clustering patterns consistently identify LCZ-8 complexes as the primary drivers of peak summer surface heating across all three cold-climate cities. The strength of this effect increases with building footprint size and with spatial aggregation of large impervious parcels, particularly where vegetative buffers are sparse.

### 3.2. Statistical validation of building-scale thermal effects

Statistical analysis confirms the strong influence of building footprint size on summer surface temperatures. One-way ANOVA applied to normalized building-level LST values yields an F-statistic of 18.82 (*p* < 0.001), indicating that differences between building size categories far exceed within-category variability. Building footprint size, therefore, has a strong and statistically robust effect on surface temperature.

Post-hoc Tukey HSD tests identify the specific contrasts driving this result (Table 11). Extra-large buildings are significantly warmer than small (Δ = 0.19, *p* < 0.001) and medium buildings (Δ = 0.16, *p* < 0.001), and large buildings are significantly warmer than small buildings (Δ = 0.09, *p* = 0.023). By contrast, no significant difference is observed between small and medium buildings, which together form a relatively uniform, cooler baseline. Importantly, a significant scale effect exists within LCZ 8 itself: extra-large buildings are warmer than large buildings (Δ = 0.10, *p* = 0.020), demonstrating that LCZ 8 is not thermally homogeneous and that footprint size modulates the intensity of surface heating.

Although statistical tests were conducted on normalized values to enable comparison across cities, the physical magnitude of these differences is substantial. As shown in Table 3, the absolute temperature gap between extra-large and small buildings ranges from 3–4°C in Fairbanks and Nadym, and from 2–3°C in Tromsø. Effect-size metrics further confirm the strength of these contrasts: for example, the small-–extra-large comparison in Fairbanks yields Cohen’s d ≈ 3.0, which is considered extremely large. Because Cohen’s d is independent of sample size, these results demonstrate that the observed thermal contrasts are genuine and not an artifact of unequal group sizes.

Overall, the statistical results align closely with the spatial hotspot patterns. Together, they provide robust evidence that LCZ 8 structures are the principal contributors to localized summer surface heating in cold-climate cities and that the magnitude of this effect scales with building footprint size.

## 4. Discussion

The results provide quantitative confirmation that LCZ 8 plays a dominant role in shaping summer surface temperature patterns in cold-climate cities. This discussion is based directly on the quantitative results presented above. Interpretations rely on observed differences in land surface temperature across LCZ classes and building footprint categories, as well as statistically significant hotspot patterns. Broader climatic or urban context factors are mentioned only to help interpret these empirical results, not to infer causal mechanisms beyond the available data. Accordingly, the discussion emphasizes robust, morphology-driven spatial contrasts rather than speculative or process-level explanations.

While Arctic and sub-Arctic summers are generally perceived as thermally benign, the observed magnitude and spatial concentration of LCZ 8-related surface heating indicate that localized overheating is both real and systematic. These findings should not be interpreted as evidence that northern cities require the same heat adaptation strategies as warm-climate cities. Rather, they call for a re-evaluation of how localized warming interacts with infrastructure performance, seasonal comfort, and patterns of outdoor activity in high-latitude urban environments.

In most Arctic cities, summer warmth is not inherently undesirable and may even enhance outdoor social life. Informal observations and ethnographic studies indicate that residents often seek sun-exposed, thermally comfortable microsites for leisure activities, markets, and cultural events, including parking areas and forecourts of large commercial complexes. At the same time, persistent surface warming over large impervious areas may have long-term consequences for infrastructure, particularly where development occurs on permafrost. Even modest increases in summer surface temperature can accelerate permafrost degradation and increase maintenance costs and structural risk over decadal timescales [5,7,23,52]. The relevance of LCZ 8, therefore, lies not only in heat-stress mitigation but also in its broader implications for urban function, seasonal use, and environmental resilience.

### 4.1. Influence of building footprint size on surface temperature

A central result of this study is that, in Arctic and sub-Arctic cities, horizontal building scale exerts a stronger control on summer surface temperature than vertical density. This contrasts with classical urban heat island theory, which associates maximum warming with compact high-rise morphologies typical of LCZ 1 and LCZ 2 in temperate and tropical cities [15]. In Fairbanks, Tromsø, and Nadym, the warmest surfaces are consistently associated with large and extra-large buildings characteristic of LCZ 8, often exceeding the surrounding urban fabric by 3–4°C. Similarly, Li et al. (2024) found the highest LST (with up to 4°C LST anomaly) in Yakutsk, collocated with the large buildings of Urasa Moll at the north and the Expo area at the south.

This pattern aligns with recent work showing that footprint area, floor-area ratio, and impervious surface fraction can outperform building height as predictors of surface warming [54]. In cold climates, the effect is amplified by construction practices optimized for winter performance: thick insulation, massive envelopes, and low surface porosity reduce heat loss during winter but also slow cooling during summer [55]. As a result, large buildings retain heat efficiently even under relatively modest solar forcing.

The airport case illustrates the importance of morphology and context. In Tromsø, the airport runway is extensive and paved, yet it does not stand out as a dominant feature. In contrast, clusters of large buildings—such as the university campus and industrial water-front—create clear thermal anomalies despite maritime moderation. In Fairbanks and Nadym, airports do register as hotspots, reflecting continental climates, sparse vegetation, and weaker landscape moderation. This distinction supports the view that LCZ behavior cannot be inferred from surface type alone: terminals and hangars behave thermally as LCZ 8, while runways and aprons more closely resemble LCZ E, and their impact depends strongly on aggregation and surrounding context [56].

Hotspot analysis reinforces these findings. In Fairbanks (and similarly in Yakutsk, according to Li et al. [53]), LCZ-8 hotspots appear as fragmented clusters embedded within a cooler residential and vegetated matrix. In contrast, Tromsø exhibits a more compact urban core where LCZ 8 overlaps with LCZ 2, concentrating thermal extremes. Nadym shows the most centralized pattern: the city itself forms a regional hotspot relative to the surrounding tundra, with the strongest intra-urban extremes concentrated in the LCZ 8 industrial core. These contrasts demonstrate that LCZ 8 dominance manifests differently across urban layouts but consistently drives the highest summer surface temperatures in cold-climate cities.

Addressing Research Question 2, the results demonstrate that both the intensity and spatial clustering of summer thermal hotspots scale systematically with building footprint size and with the spatial aggregation of large impervious parcels. Hotspot analyses show that isolated large buildings generate localized anomalies, whereas spatially continuous clusters of large and extra-large buildings produce persistent, district-scale hotspot zones. This pattern is consistent across all three cities and confirms that LCZ-8 thermal behavior is not solely a function of land-cover type, but of horizontal scale and spatial concentration.

### 4.2. Urban form, materials, and seasonal microclimatic modulation

Large low-rise buildings amplify summer surface heating through both scale and surface properties. Extensive asphalt parking areas, metal roofs, and concrete façades exhibit high thermal inertia, storing heat during the day and releasing it slowly at night [45,57]. In the LCZ 8 districts, vegetation is typically sparse, suppressing evapotranspiration—one of the most effective natural cooling mechanisms [46]. As a result, thermal contrasts between LCZ 8 and surrounding urban zones remain sharp even under weak synoptic forcing.

Vegetation mitigates these effects through shading and latent heat flux, consistent with global evidence on the cooling role of urban green infrastructure [47,48]. However, in Arctic cities, vegetation density is constrained by climate, soil conditions, and snow management practices, and green infrastructure is often secondary to mobility and maintenance requirements [58,59]. Consequently, green corridors such as those in Tromsø provide localized cooling but cannot fully offset heating generated by dense LCZ 8 clusters. Seasonality further complicates interpretation. Snow cover increases albedo and suppresses urban heat islands during winter [60], while its absence in summer exposes large impervious surfaces to continuous solar input. This seasonal inversion of LST anomalies was further accentuated in the global study by Sismanidis et al. [61]. For the urban-scale surface heat island intensity, the study reported that, for low LST (< 300 K), the daytime LST anomaly in cold-climate cities is nearly constant at about 1 K. For high LST (> 300 K), the urban LST anomaly increases by 5–6 K. The corresponding nighttime anomalies are rather flat at about 1–2 K, which could be explained by long daytime at high latitudes. Globally, the inter-quartile range was found to be [0.2 K, 2.7 K] for daytime and [0.4 K, 2.4 K] nighttime LST anomalies in the Dfb-climate cities. On the other side, detailed in situ studies, such as e.g., Varentsov et al. [34] in Nadym, do not support the conclusions of the global studies based on remote sensing data analysis. In-situ studies emphasize the differences between dense and sparse building arrangements. High anthropogenic heat flux from building heating raises LST within dense LCZs in the cold season, whereas sparsely built LCZ-8 does not show this effect. This explanation is supported by the quantitative heat budget decomposition study by Guo et al. [62]. Thus, compelling results indicate that LCZ 8 forms optimized for winter efficiency can become dominant sources of summer surface heating. This dynamic remains underexplored in LCZ-based research but is particularly relevant for Arctic urbanism.

### 4.3. Are heat-mitigation strategies compatible with high-latitude urban needs?

The results suggest that in cold-climate cities, heat mitigation may not constitute a universal planning priority. Rather than addressing summer warming as a citywide problem, interventions may be warranted only where localized LCZ-8-related heating intersects with infrastructure vulnerability, material performance, or intensive public use. In Arctic and sub-Arctic contexts, summer warmth is often not inherently problematic and may enhance outdoor comfort and social activity during short warm seasons [1,4]. Interventions that indiscriminately suppress surface warming therefore risk conflicting with seasonal use patterns, cultural preferences for sun-exposed space, and long-standing winter-oriented design priorities [1].

At the same time, LCZ-8-related overheating is highly localized, concentrated around aggregated large-footprint, low-rise building complexes rather than uniformly distributed across the urban fabric. In Nadym, LCZ 8 forms a centralized thermal core relative to the surrounding tundra, where repeated summer warming may contribute to permafrost degradation and long-term infrastructure risk [23,63,64]. In Fairbanks, extra-large buildings reach surface temperatures exceeding 38°C, indicating conditions where excessive heat storage may negatively affect material performance and local comfort, consistent with findings from other cold-region cities [32]. These findings support a targeted rather than uniform approach to thermal intervention in cold-climate cities.

Material-based strategies are particularly compatible with the needs of high-latitude urban areas. Higher-albedo roofs and pavements can reduce excessive heat storage while preserving solar access that remains critical for winter performance [44,45,65]. Observations from Fairbanks suggest that lighter-colored roofs on large buildings are already associated with lower surface temperatures, indicating that albedo-based measures can be effective even in Arctic contexts. By contrast, extensive greening—often central to warm-climate heat-mitigation strategies—may deliver limited or inconsistent performance in northern cities due to short growing seasons, snow management requirements, and maintenance constraints [58,59].

Urban form also plays an important role. Ventilation corridors, frequently designed to improve air quality, can contribute to convective cooling during episodic summer heat, particularly in compact cities such as Tromsø [31]. Conversely, the relatively contained urban form of cities like Nadym—shaped by Soviet-era planning and constrained expansion—has limited the proliferation of large impervious surfaces, indirectly restricting heat-prone development [24,25]. These examples illustrate that planning decisions affecting building form, spatial organization, and land-use intensity can influence thermal outcomes even in the absence of explicit heat-mitigation policies.

At high latitudes, solar access remains a central planning objective. Designs that maximize winter solar gain under conditions of short daylight and low sun angles may inadvertently intensify summer surface heating in unshaded LCZ 8 forecourts, parking areas, and plazas [65]. Seasonally adaptive strategies—such as deciduous vegetation, lightweight canopies, and low-mass, high-albedo surfaces—offer a means of balancing winter efficiency with summer comfort [1,47]. Rather than importing mitigation concepts wholesale from warm climates, northern cities may benefit from a dual-season approach that preserves beneficial warmth while avoiding excessive heat accumulation in large impervious districts [32,60].

### 4.4. LCZ 8 as an opportunity for indoor–outdoor public-space integration in cold climates

The interpretation of LCZ 8 in this section is motivated by two empirical observations from this study. First, LCZ-8-related surface warming is spatially concentrated rather than citywide. Second, these zones systematically coincide with large-footprint buildings that already function as urban destinations, such as shopping centers, campuses, and transport hubs. Given these characteristics, the LCZ 8 areas represent not only thermal entities but also functionally important urban spaces.

To interpret the broader implications of these findings, we draw on documented practices from other Arctic and sub-Arctic cities, where comparable large-building districts have been integrated into everyday public life through seasonally adaptive indoor–outdoor design. These examples are used to contextualize, rather than generalize, the observed thermal behavior of LCZ 8 within established patterns of cold-climate urban use. Rather than being interpreted solely as negative thermal anomalies, LCZ 8 districts can therefore be understood as seasonally distinctive urban environments whose localized summer warmth may support recreational use, outdoor activity, and social interaction during short warm periods, provided that excessive heat accumulation is managed.

Beyond questions of mitigation, the spatial concentration of LCZ-8-related warming supports an alternative interpretation of large low-rise districts in cold-climate cities. Because elevated surface temperatures are largely confined to specific parcels and clusters, LCZ 8 areas can function as seasonally advantageous microclimatic zones rather than as uniformly problematic hotspots. In Arctic and sub-Arctic contexts—where substantial populations live permanently on permafrost and depend on year-round urban functionality—such zones represent structurally important components of everyday urban life rather than marginal or residual spaces [51].

In all three study cities, LCZ 8 coincides with shopping centers, campuses, transport hubs, and other destinations that already attract regular foot traffic, indicating latent potential for integrating indoor–outdoor public spaces. From a microclimatic perspective, large-footprint, low-rise districts actively shape local thermal conditions through their horizontal scale, surface materials, and density configu-ration, influencing solar exposure, wind shelter, and heat storage even in cold climates [56,60]. These properties help explain why LCZ 8 areas frequently operate as transitional environments between indoor and outdoor public life.

Although the thermal anomalies analysed in this study occur in summer, examples from winter-city planning are introduced here to illustrate how large-footprint building districts function as year-round public-space anchors in cold-climate cities, where seasonal continuity—rather than single-season optimisation—defines urban usability. Experience from winter-city planning in northern Europe and northern North America demonstrates that successful public-space systems in cold climates rely less on eliminating thermal contrasts than on maintaining continuity between indoor and outdoor environments [1, 64]. In Reykjavík, geothermal snow-melt systems integrated into the public realm keep key pedestrian routes operational throughout winter, effectively transforming streets and sidewalks into reliable extensions of indoor destinations [65,66]. Similarly, winter-city practice in Oulu highlights the importance of network continuity over iconic architectural intervention, where consistent winter maintenance, snow management, lighting, and surface treatment enable central pedestrian zones to function as stable public anchors across seasons [67–69]. These examples underscore that the usability of outdoor space in cold climates depends as much on governance and maintenance regimes as on localized microclimatic optimization.

A complementary planning approach is evident in the redevelopment of civic centers in northern cities such as Kiruna, where large-footprint public buildings are intentionally paired with outdoor civic spaces designed around wind shelter, enclosure, and solar access [70]. This “anchor-and-forecourt” configuration demonstrates that LCZ-8-scale buildings need not generate residual or leftover space; when deliberately designed, they can structure outdoor rooms that operate as extensions of indoor public life even under harsh climatic conditions [71–73].

Finally, governance practices in places such as Whitehorse underline that indoor publicness itself can be treated as urban infrastructure in cold climates. Seasonal warming centers and other publicly accessible indoor facilities function as nodes within broader social infrastructure and access networks, supporting social interaction and mobility when outdoor conditions are limiting [74–77]. This perspective is particularly relevant for LCZ 8 districts, where many large buildings are privately owned yet functionally public, and where planning frameworks can explicitly recognize indoor–outdoor continuity as a component of urban resilience.

Taken together, these insights support a reframing of LCZ 8 in cold-climate cities. Rather than viewing large low-rise districts exclusively as thermal liabilities to be mitigated, they can be understood as strategic interfaces where microclimate, infrastructure, governance, and public life intersect. Planning approaches that prioritize continuity, seasonal adaptability, and spatial targeting—rather than uniform heat suppression—are therefore better aligned with the magnitude, localization, and functional role of LCZ-8-related warming observed in Arctic and sub-Arctic cities.

### 4.5. Uncertainty and limitations

This study is subject to several limitations related to temporal sampling, reliance on satellite-derived surface temperatures, and the absence of process-level attribution, which are clarified here to support transparent interpretation of the results.

Land surface temperature (LST) derived from Landsat thermal infrared data is subject to unavoidable uncertainties arising from surface emissivity estimation, atmospheric water vapor effects, and sensor characteristics. Numerous validation studies demonstrate that, under clear-sky conditions, Landsat-based single-channel and split-window retrieval algorithms typically achieve mean biases below 1 K and root-mean-square errors of 1–3 K when evaluated against dense ground-based geosensor networks and radiometric reference sites [38–41]. Independent validations of Landsat-8 TIRS Band 10 across diverse surface types in the conterminous United States confirm stable performance and error magnitudes well below the spatial temperature contrasts commonly observed within urban environments [39].

Although site-specific in situ validation data were not available for the study cities, the thermal anomalies identified here—especially the 3–6°C contrasts between extra-large LCZ-8 structures and surrounding urban fabric—substantially exceed reported retrieval uncertainties and exhibit strong spatial coherence across multiple independent summer scenes. Consequently, this analysis emphasizes relative spatial patterns and inter-class thermal contrasts rather than absolute temperature values, an approach widely adopted in LCZ-based urban climate studies and considered robust for comparative assessment in data-sparse Arctic environments [38,39,41].

A further limitation is that the analysis relies on satellite-derived surface temperatures and GIS-based morphological descriptors, which do not resolve the physical processes governing urban heat exchange. The available datasets do not allow explicit quantification of contributions from building energy use, insulation performance, material thermal inertia, sky-view factor, shading geometry, or local ventilation and airflow regimes. As a result, the study does not attempt to mechanistically partition the individual heat-contributing processes within LCZ 8. The reported relationships should therefore be interpreted as empirical associations between urban form and surface thermal response, rather than as causal attribution to specific physical mechanisms.

Finally, temporal sampling is limited to a small number of clear-sky summer scenes per city. While these scenes capture stable spatial thermal structures, they do not represent short-term weather variability, diurnal extremes, or rare heat events. Future research should integrate high-resolution numerical modelling approaches—such as surface energy balance modelling, sky-view-factor-based radiative analysis, computational fluid dynamics (CFD), or mesoscale urban climate models—with scenario-based testing of material properties, vegetation configurations, and urban layout. Such approaches would enable process-based evaluation of LCZ-8 thermal behavior and improve transferability across cold-climate urban contexts.

## 5. Conclusions

This study demonstrates that in cold-climate cities, large-footprint, low-rise development is a primary driver of summer surface thermal extremes. Using Landsat-derived land surface temperature (LST), detailed building footprint data, and spatial hotspot analysis for Fairbanks, Tromsø, and Nadym, we show that summer surface warming in Arctic and sub-Arctic cities is governed less by building height or overall urban density than by horizontal scale, spatial aggregation, and the dominance of impervious materials. This finding fundamentally distinguishes high-latitude urban thermal behavior from patterns commonly reported in warm and temperate cities and confirms horizontal urban morphology as a critical control on surface heating in northern environments.

The main conclusions are as follows:

1. Building footprint size strongly controls summer surface heating in cold-climate cities. Across all three cities, large and extra-large buildings consistently exhibit the highest summer LST values. Absolute temperature differences between extra-large and small buildings reach approximately 3–4°C in Fairbanks and Nadym and 2–3°C in Tromsø, substantially exceeding known Landsat retrieval uncertainties. Statistical analysis confirms that these differences are robust and scale-dependent, including within LCZ 8 itself, demonstrating that LCZ-8 districts are not thermally homogeneous but internally structured by footprint size.
2. Thermal hotspot intensity and spatial clustering increase with the spatial aggregation of large-footprint buildings. Beyond individual buildings, hotspot intensity and clustering increase systematically as large and extra-large buildings become spatially aggregated. Getis–Ord Gi* analysis reveals that isolated large buildings generate localized anomalies, whereas spatially continuous LCZ-8 districts form persistent, statistically significant hotspot clusters. This directly addresses Research Question 2 and confirms that LCZ-8 thermal behavior depends on both horizontal scale and spatial configuration.
3. LCZ 8 consistently defines the upper bound of urban summer surface temperatures. Very large, impervious building complexes in LCZ 8 exhibit the highest mean and maximum LST values across all three cities, despite differences in climate, urban layout, and development history. These hotspots are not anomalous outliers but systematic expressions of contemporary horizontal urban form.
4. LCZ-8-related warming is localized rather than citywide, implying targeted planning responses. Thermal anomalies are spatially confined to large impervious districts rather than evenly distributed across the urban fabric. This indicates that heat-related interventions in cold-climate cities should focus on specific large-footprint developments where overheating intersects with infrastructure vulnerability or intensive public use.
5. Localized summer warming in LCZ 8 has both risks and potential benefits in cold climates. While excessive surface heating may exacerbate material stress and permafrost degradation, moderate localized warming can enhance outdoor comfort and seasonal public life during short Arctic summers. This dual role underscores the need for scale-sensitive and seasonally adaptive urban design strategies rather than the wholesale transfer of warm-climate heat-mitigation paradigms.

Overall, this study calls for a shift in how urban heat is conceptualized and managed in cold regions. LCZ 8 should be understood not as a marginal or residual urban form, but as a key interface between climate processes, horizontal urban morphology, and everyday public life. Explicitly incorporating the horizontal dimension of urban development into urban climate theory and planning practice is essential for creating resilient, livable, and climate-responsive Arctic and sub-Arctic cities.

## Figures and Tables

**Table 1:** Details of Landsat-8 scenes used for LST analysis (three cloud-free summer scenes per city, June–August 2013–2025).

City	Scene ID	Acquisition Date	Cloud Cover
**Fairbanks**	LC08_070015_20210701	2021-07-01	0.84 %
	LC08_067016_20180723	2018-07-23	0.31 %
	LC08_070014_20160617	2016-06-17	0.29 %
**Tromsø**	LC08_197011_20220730	2022-07-30	0.75 %
	LC08_199011_20180717	2018-07-17	1.54 %
	LC08_199011_20170612	2017-06-12	1.63 %
**Nadym**	LC08_161014_20240707	2024-07-07	0.00 %
	LC08_160014_20180630	2018-06-30	0.01 %
	LC08_159014_20170706	2017-07-06	0.02 %

**Table 2:** Number of buildings in each size class.

City	Small (<1,000 m^2^)	Medium (1,000–5,000 m^2^)	Large (5,000–10,000 m^2^)	Extra-large (>10,000 m^2^)	Total
Fairbanks	3,021	1,144	213	46	4,424
Tromsø	6,812	1,738	317	98	8,965
Nadym	542	341	79	48	1,010

**Table 3:** Building-level maximum LST (°C): mean and SD by footprint class.

	Fairbanks		Tromsø		Nadym	
	Mean	SD	Mean	SD	Mean	SD
Extra-large	33.9	2.8	24.3	2.1	26.7	2.7
Large	31.4	1.3	22.5	1.1	25.6	1.0
Medium	30.3	1.4	21.0	1.5	24.5	1.0
Small	29.6	1.4	20.3	1.4	24.2	1.0

**Table 4:** Summary of LST statistics across Local Climate Zones (LCZs) for Fairbanks, Tromsø, and Nadym.

City	LCZ Name	Min (°C)	Max (°C)	Mean (°C)
**Fairbanks**	5 Open midrise	24.4	31.4	28.7
	6 Open low-rise	18.3	32.9	27.2
	**8 Large low-rise**	**19.7**	**38.1**	**29.2**
	A Dense trees	18.4	33.4	24.8
	B Scattered trees	19	32.6	25.7
	D Low plants	18.3	35.9	27
	E/F Bare rock	18.1	32.8	29
**Tromsø**	2 Compact midrise	17.7	22.3	21.3
	5 Open midrise	16.4	24.4	20.3
	6 Open low-rise	12.5	23.5	19.3
	**8 Large low-rise**	**13.4**	**26.7**	**20.5**
	10 Heavy industry	15.8	21.5	20
	A Dense trees	13.2	21	16.8
	B Scattered trees	15.4	20	18
	D Low plants	12	22.3	18.2
	E/F Bare rock	18.9	21.5	20.6
**Nadym**	5 Open midrise	21	26.2	24.2
	6 Open low-rise	18.8	26.7	23.5
	**8 Large low-rise**	**21.1**	**31.1**	**25**
	9 Sparsely built	20.7	25.4	23.2
	A Dense trees	20.4	24.9	23.4
	E/F Bare rock	19.3	26.1	24

**Table 5:** Building statistics by LCZ for Fairbanks.

LCZ	Building Count	Total Area (m+)	Mean Area (m^2^)	% Large + XL
5	525	103,643	197.4	3.0 %
6	3,439	823,866	239.6	7.6 %
8	1,682	1,196,143	711.1	29.1 %

**Table 6:** Hotspot statistics by LCZ for Fairbanks.

LCZ	Building Count	% Hotspot	% Coldspot	% Neutral
5	525	89.5 %	0.2 %	10.3 %
6	3,439	74.8 %	15.0 %	10.1 %
8	1,682	88.5 %	4.9 %	6.7 %

**Table 7:** Distribution of building sizes across LCZs for Tromsø.

LCZ	Building Count	Total Area (m^2^)	Mean Area (m^2^)	% Large + XL
6	7,630	1,314,300	172.3	4.6 %
5	963	218,425	226.8	8.2 %
8	562	549,700	978.1	41.8 %
2	310	109,675	353.8	15.2 %

**Table 8:** Hotspot statistics by LCZ in Tromsø.

LCZ	Building Count	Mean LST (°C)	% Hotspot	% Coldspot	% Neutral
2	310	21.3	17.4 %	20.0 %	62.6 %
5	963	20.2	13.9 %	23.4 %	62.7 %
6	7,630	19.3	13.6 %	28.2 %	58.2 %
8	562	20.5	40.9 %	11.2 %	47.9 %

**Table 9:** Building statistics by LCZ for Nadym.

LCZ	Building Count	Total Area (m^2^)	Mean Area (m^2^)	% Large + XL
5	419	373,536.6	891.5	64.9 %
6	651	435,548.9	669.0	59.1 %
8	224	320,797.1	1432.1	62.1 %
9	16	11,990.9	749.4	75.0 %

**Table 10:** Hotspot statistics by LCZ for Nadym.

LCZ	Building Count	Mean LST (°C)	% Hotspot	% Coldspot	% Neutral
5	206	24.3	40.8 %	7.3 %	51.9 %
6	302	23.7	11.3 %	42.1 %	46.7 %
8	140	25.0	82.9 %	5.0 %	12.1 %
9	8	23.7	0.0 %	12.5 %	87.5 %

**Table 11:** Pairwise comparisons of mean normalized LST by urban form category.

Comparison (warmer vs. cooler category)	Associated LCZ comparison	Mean difference (Δ)	p-value	Significance
Extra large vs. small	LCZ 8 vs. mixed urban fabric	0.19	<0.001	Significant
Extra large vs. medium	LCZ 8 vs. mixed urban fabric	0.16	<0.001	Significant
Extra large vs. large	Within LCZ 8 (testing scale effect)	0.10	0.020	Significant
Large vs. small	LCZ 8 vs. mixed urban fabric	0.09	0.023	Significant
Medium vs. small	Mixed urban vs. mixed urban	0.03	0.754	Not Significant
Large vs. medium	LCZ 8 vs. mixed urban fabric	0.07	0.128	Not Significant

## Data Availability

Data will be made available on request.
